# Association Between Serum Cobalt and Manganese Levels with Insulin Resistance in Overweight and Obese Mexican Women

**DOI:** 10.3390/healthcare13192511

**Published:** 2025-10-02

**Authors:** Jacqueline Soto-Sánchez, Héctor Hernández-Mendoza, Gilberto Garza-Treviño, Lorena García-Morales, Bertha Irene Juárez Flores, Andrea Arreguín-Coronado, Luis Cesar Vázquez-Vázquez, María Judith Rios-Lugo

**Affiliations:** 1 Sección de Estudios de Posgrado e Investigación, Escuela Nacional de Medicina y Homeopatía, Instituto Politécnico Nacional, Av. Guillermo Massieu Helguera 239, Ciudad de México 07320, Mexico; jasotos@ipn.mx (J.S.-S.); ggarzat@ipn.mx (G.G.-T.); 2 Instituto de Investigación de Zonas Desérticas, Universidad Autónoma de San Luis Potosí, Altair 200, San Luis Potosí 78377, Mexico; hector.mendoza@uaslp.mx (H.H.-M.); berthajf@uaslp.mx (B.I.J.F.); 3 Laboratorio Estatal de Salud Publica, Comisión Estatal para la Protección Contra Riesgos Sanitarios, Begonias 180, San Luis Potosí 78380, Mexico; 4 Sección de Estudios de Posgrado e Investigación, Escuela Superior de Enfermería y Obstetricia, Instituto Politécnico Nacional, Casco de Calle Manuel M. Carpio S/N, Ciudad de México 11340, Mexico; 5 Facultad de Enfermería y Nutrición, Universidad Autónoma de San Luis Potosí, Avda. Niño Artillero 130, San Luis Potosí 78210, Mexico; andrea.arreguin@uaslp.mx; 6 Sección de Medicina Molecular y Traslacional, Centro de Investigación en Ciencias de la Salud y Biomedicina, Universidad Autónoma de San Luis Potosí, Avda Sierra Leona 550, San Luis Potosí 78210, Mexico; luiscesarvzv@gmail.com

**Keywords:** insulin resistance, fasting insulin, fasting glucose, cobalt, manganese, overweight and obesity

## Abstract

**Background:** Insulin resistance (IR) is common in overweight or obese individuals. Dysregulation of trace elements such as cobalt (Co) and manganese (Mn) has been associated with obesity and IR markers in individuals with diabetes. However, their role in non-diabetic states is less understood. **Objective:** This study aimed to analyze the association between serum Co and Mn levels and IR in overweight and obese women without diabetes. **Methods:** A total of 112 overweight or obese women were evaluated for their anthropometric, metabolic, and biochemical characteristics. To estimate IR, the homeostatic model assessment of insulin resistance (HOMA-IR), quantitative insulin sensitivity check index (QUICKI), triglyceride–glucose index (TyG), and triglyceride–glucose–body mass index (TyG-BMI) were calculated. Serum Co and Mn concentrations were quantified by inductively coupled plasma mass spectrometry (ICP-MS). **Results:** Our results show that 77% of participants exhibited central fat accumulation and a high prevalence of IR. Fasting insulin (FINS), HOMA-IR, and TyG-BMI were significantly higher in obese women, while adiponectin (Adpn) was lower. Moreover, Co was inversely associated with FINS (*p* = 0.003) and HOMA-IR (*p* = 0.011), and positively associated with QUICKI (*p* = 0.011) in obese women. In contrast, serum Mn levels showed negative correlations with fasting glucose (FG) (*p* = 0.021) and the TyG index (*p* = 0.048) in overweight women. **Conclusions:** Co serum levels were positively associated with FG and QUICKI and negatively associated with FINS and HOMA-IR in the obese group. Mn showed negative associations with FG and the TyG index, suggesting that these trace elements may play a role in the IR in people with obesity.

## 1. Introduction

Obesity is a chronic, multifactorial disease characterized by the abnormal or excessive accumulation of white adipose tissue, and it has reached pandemic proportions. It is estimated that by 2030, up to 1.13 billion adults worldwide are predicted to be obese or overweight [1]. At the national level, in Mexico, the prevalence of overweight and obesity in adults is 38.3% and 36.9%, respectively [2]. On the other hand, this disorder is often accompanied by metabolic alterations such as insulin resistance (IR), fasting hyperglycemia, impaired glucose tolerance, and dyslipidemia, which together increase the risk of developing type 2 diabetes mellitus (T2DM) and cardiovascular disease [3].

Under physiological conditions, insulin promotes glucose uptake in insulin-dependent tissues, such as the myocardium and skeletal muscle, thereby contributing to glycemic homeostasis [4]. However, in states of IR, these tissues show a decreased response to insulin action, despite elevated plasma concentrations [5]. In addition to insulin, another key adipokine in regulating sensitivity to this hormone is adiponectin (Adpn). This hormone is secreted by adipose tissue and promotes insulin response in peripheral tissues, improves glucose tolerance, and stimulates fatty acid oxidation [6]. Its concentrations are inversely correlated with the degree of obesity and the amount of adipose tissue [7].

Trace elements have been shown to play essential roles in various cellular metabolic processes, acting as enzyme cofactors and modulators of key biochemical pathways. In particular, they have been identified as playing a role in the regulating insulin signaling, thereby influencing cellular sensitivity and glucose homeostasis [3,8]. Among these, cobalt (Co) stands out, an essential trace element mainly due to its presence as the central atom of vitamin B_12_, which is obtained directly from dietary sources and plays an indispensable role in cellular metabolism, including DNA synthesis, erythropoiesis, and central nervous system function [9,10]. Various chemical forms of Co, such as cobalt chloride (CoCl_2_) and cobalt protoporphyrin (CoPP), have demonstrated beneficial effects on activities of antioxidant enzymes, lipid metabolism, and insulin signaling in animal models of obesity and diabetes [11,12]. For example, CoCl_2_ administration normalized adipocyte size, reduced plasma triglyceride (TG) and free fatty acid (FFA) levels, and increased Adpn expression [13].

Another essential trace element is manganese (Mn), which is indispensable as a cofactor for various Mn-dependent enzymes involved in multiple biological processes [14]. This element contributes to the regulation of glucose metabolism by activating signaling pathways shared with those of insulin. Its deficiency has been associated with glucose intolerance and decreased pancreatic insulin secretion [15]. In addition, Mn supplementation improves these parameters and stimulates the activity of manganese-dependent superoxide dismutase (MnSOD), a key enzyme that eliminates reactive oxygen species, which are abundant in T2DM [16].

Population studies have reported associations between Co and Mn levels and prediabetic and diabetic [17,18,19]. Additionally, its relationship with glycemic parameters, such as serum glucose levels and the homeostatic model assessment of insulin resistance (HOMA-IR), has been explored [20,21,22]. However, some studies have evaluated the association between serum Co and Mn levels and IR markers in non-diabetic populations [23,24]. Considering that an imbalance in trace element levels is often observed in overweight or obese individuals, and that Mn and Co status may differ in these populations [25,26], it is possible that their association with IR also varies. Therefore, our study has focused on the assessing the association between serum Mn and Co levels and markers of IR in overweight and obese women, with the aim of better understanding the role of these trace elements in insulin homeostasis and elucidating the impact of excess adiposity on IR.

## 2. Materials and Methods

### 2.1. Study Design

This cross-sectional study was conducted between 2022 and 2023 in Mexico City. The study population consisted of adult women, aged between 18 and 44 years, with a body mass index (BMI) of 25 kg/m^2^ or greater. Participants were recruited through announcements and invitations on social media platforms such as Facebook, Instagram, and X, as well as local advertisements at the Escuela Nacional de Medicina y Homeopatía, part of the National Polytechnic Institute in Mexico City. Women who were pregnant, breastfeeding, athletes, diagnosed with diabetes mellitus or high blood pressure, or who were undergoing treatment with pharmacological, lipid-lowering drugs, hypoglycemic drugs, oral contraceptives, or vitamin and mineral supplements during the three months before their participation were excluded.

### 2.2. Ethics Approval

All procedures performed in studies involving human participants complied with the ethical standards of the institutional review board and the 1964 Declaration of Helsinki, as well as its subsequent amendments or comparable ethical standards. The study was approved by the Bioethics Committee of the National School of Biological Sciences of the National Polytechnic Institute (approval registration: ENCB/CEI/064/2021) (ENCB/CEI/064/2021) on 3 November 2021, and the official document was issued in January 2022. Additionally, all individual participants provided written informed consent in accordance with institutional and ethical guidelines.

### 2.3. Anthropometric Assessment

Weight and height were measured using a calibrated stadiometer (SECA), with the subject wearing light clothing and without shoes, to an accuracy of 0.1 kg and 0.1 cm, respectively. Waist circumference (WC) and hip circumference (HC) were measured at the midpoint between the lower edge of the rib cage and the iliac crest, and around the widest part of the buttocks, respectively, with an accuracy of 0.5 cm, using a non-stretchable tape measure. All anthropometric assessments were conducted using properly calibrated equipment and following standardized procedures. To reduce inter-observer variability, all evaluators underwent prior training and used consistent measurement protocols. Obesity indices, including BMI, waist-to-hip ratio (WHR), and waist-to-height ratio (WHR), were then calculated. BMI was classified into two categories: overweight (25.0–29.9 kg/m^2^) and obese (≥30.0 kg/m^2^).

### 2.4. Biochemical Assessment

After fasting for 8 to 12 h, blood samples were collected from each participant. The samples were then centrifuged at 3000× *g* for 10 min to obtain serum. Samples were divided into aliquots and stored at 4 °C and −80 °C until analysis. Fasting glucose (FG), TG, and total cholesterol (TC) were quantified using a clinical chemistry analyzer (EasyKem Plus, KONTROLab, Naples, Italy). Adpn and fasting insulin (FINS) concentrations were determined using an enzyme-linked immunosorbent assay (ELISA) (Invitrogen-Thermo Fisher Scientific, Waltham, MA, USA), following the manufacturer’s instructions. Sample absorbance was taken on a microplate reader at 450 nm (WHYM201, Poweam, Nanjing, China). All determinations were performed in duplicate.

### 2.5. Insulin Resistance Assessment

IR was assessed using the following indices: HOMA-IR = insulin (µU/mL) × FG (mg/dL)/405. The quantitative insulin sensitivity check index (QUICKI) was calculated from FG and FINS levels using the formula: QUICKI = 1/(log [FINS in µU/mL] + log [FG in mg/dL]). The triglyceride–glucose index (TyG) was obtained using the formula: Ln (TG [mg/dL] × FG [mg/dL]/2), and the TyG-body mass index (TyG-BMI) was calculated as TyG-BMI = BMI × TyG index. Participants with HOMA-IR levels > 2.5, QUICKI ≤ 0.33, TyG index ≥ 4.68, and TyG-BMI ≥ 227 were considered to have IR. These cutoff values for HOMA-IR, QUICKI, TyG, and TyG-BMI were taken from previous studies in Mexican adults without diabetes [27,28,29], as no population-specific thresholds are available for young Mexican women.

### 2.6. Co and Mn Analysis

The sample treatment followed the protocol proposed by Hernández-Mendoza et al. [30], with additional quality control procedures to ensure accuracy and minimize contamination. All samples were traced with an internal standard [Indium (In); 50 μg/L] and digested in teflon vessels using a closed-vessel microwave system (MARS 6, CEM corporation) with 5 mL of HNO_3_ for 15 min at 220 °C. After digestion, samples were evaporated in teflon containers, and the residues were diluted to a final volume of 5 mL with 2% *v*/*v* HNO_3_. All volumetric measurements were performed with class A glassware.

Cross-contamination during digestion was evaluated by including reagent blanks and cleaning blanks throughout the analytical sequence. The measurement sequence consisted of one cleaning blank, a calibration curve, four cleaning blanks, ten serum samples, one cleaning blank, quality-control, and one cleaning blank. This cycle was repeated across the run. This sequence has been stable in our laboratory previously. Quality control corresponded to 75 μg/L, this being a pre-preparation from certified standard dissolutions.

Co and Mn analysis was performed using Inductively Coupled Plasma Mass Spectrometry (ICP–MS iCAP Q, Thermo Scientific, Bremen, Germany) with Helium Kinetic Energy Discrimination (He KED), and a collision/reaction cell (CRC) to minimize spectral interference. Co and Mn concentrations were obtained using an external calibration curve (0.01, 0.1, 1, 5, 10, 25, 50, 100, 150, and 200 μg/L). HNO_3_ concentrated high-purity (Milestone Duopur system by Milestonesrl, Sorisole, Italy), high-purity water with 18 MΩ cm (Milli-Q^®^ system by Millipore, Burlington, MA, USA), and Co and Mn standards dissolution (High–Purity Standards, North Charleston, SC, USA) were employed in the sample preparation and analysis.

### 2.7. Statistical Analysis

The study’s data were analyzed using SPSS (version 25.0, IBM, Armonk, NY, USA). Continuous variables with non-normal distributions were expressed as medians (interquartile range, IQR), and categorical variables were presented as numbers or percentages. Differences between the overweight and obese groups were evaluated using the Mann–Whitney U test. Correlations between variables were determined using Spearman’s correlation coefficient. The regression coefficient (Beta) and 95% confidence intervals (CI) were estimated to reflect the association between Co and Mn levels with markers of IR using multiple linear regression. Natural logarithmic transformation were applied to non-normal continuous variables—specifically, WC, HP, BMI, FINS, HOMA-IR, Co and Mn—prior to regression analysis to meet model assumptions. Moreover, two models were used: Model 1, adjusted for weight, height, WC, and HP; Model 2, adjusted for WHR, WHtR, and BMI. To control for multiple comparisons, the Benjamini–Hochberg false discovery rate (FDR) correction was applied to both Spearman correlation and linear regression models when appropriate. Values of *p* < 0.05 were considered statistically significant.

## 3. Results

### 3.1. Analytical Method Validation

The analytical method demonstrated robust performance for the quantification of Mn and Co. Calibration curves for Co and Mn exhibited excellent linearity, with r^2^ values of 0.9999 and 0.9996, respectively. The limits of detection (LOD) were 0.002 μg/L for Co and 0.009 μg/L for Mn, while the limits of quantification (LOQ) were 0.02 μg/L and 0.09 μg/L, respectively. Accuracy, evaluated through internal standard recoveries, ranged from 77% to 109% –the average of Co and Mn was 93 ± 12% and 93 ± 16%, respectively, and precision was confirmed by relative standard deviations (RSD) below 3%. The Control quality used yielded results within the expected ranges, with 74.652 ± 0.559 μg/L for Co and 74.478 ± 0.770 μg/L for Mn, confirming the reliability of the determinations.

### 3.2. Nutritional Status in the Population

Our study includes 112 women with a median age of 22 and 23 for overweight and obesity, respectively. The anthropometric and metabolic characteristics of overweight and obese women are presented in Table 1. Overall, 77% of women exhibited central fat accumulation, and 100% showed cardiovascular risk according to the WHR (≥0.85) and WHtR (≥0.5) criteria. In terms of BMI, almost half of the women, 48.2%, were classified as overweight, while 51.8% of women were classified as obese. However, obese women had significantly higher values for weight, WC, HC, WHR, WHtR, and BMI (*p* < 0.001).

### 3.3. Biochemical Markers

No significant differences were found in FG, TG, and TC levels between the groups, although the values were numerically higher in the obese group. FINS levels were significantly higher in the obese women group (*p* < 0.001), while Adpn was lower in this group (*p* = 0.011). Likewise, metabolic indices related to IR showed significant differences between the groups.

### 3.4. Markers of Insulin Resistance and Trace Elements

Women with obesity exhibited significantly higher HOMA-IR values and lower QUICKI scores (*p* < 0.001) compared to those who were overweight. TyG-BMI was also markedly elevated in this group (*p* < 0.001), whereas the TyG index, although higher in obese participants, did not reach statistical significance (Table 1). A large majority of women with obesity (100%) and 66.67% of those with overweight presented abdominal obesity (WC ≥ 88 cm). Only 7.41% of overweight participants and 15.52% of those with obesity had elevated FG levels (≥100 mg/dL). Elevated FINS levels (≥25 µU/mL) were detected in 14.81% of overweight women and 41.38% of those with obesity. IR, defined by HOMA-IR ≥ 2.5, was prevalent in 98.28% of obese participants and 70.37% of those with overweight. In contrast, adequate insulin sensitivity, reflected by QUICKI values ≥ 0.33, was present in only 31.48% of the overweight group and 3.45% of the obese group. According to the TyG index cutoff (≥4.68), 31.48% of overweight women and 43.1% of those with obesity met the threshold. In contrast, elevated TyG-BMI values (≥227) were identified only in 3.45% of the obese group and none of the overweight participants (Table 2). Finally, in the overweight group, the median (IQR) Co concentration was 0.35 μg/dL (0.23–0.58 μg/dL), while in the obese group, it was 0.40 μg/dL (0.32–0.59 μg/dL). For Mn, the median (IQR) concentration was 0.20 μg/dL (0.14–0.35 μg/dL) in the overweight group and 0.30 μg/dL (0.16–0.46 μg/dL) in the obese group. Although these values were slightly higher in women with obesity, the differences did not reach statistical significance (*p* = 0.237 for Co, *p* = 0.136 for Mn). When compared with reference ranges for trace elements in serum, the observed values fall within the expected limits (Co = 0.11 to 0.50 μg/dL; Mn = 0.04 to 0.50 μg/dL), with some individuals, particularly in the obese group, presenting values near the upper end of these ranges (Table 1).

### 3.5. Analysis of Correlations

Our results show that a negative and statistically significant correlation was observed between Co levels and WC (r = –0.270; *p* = 0.04) in the overweight group; however, this association did not remain significant after the FDR correction. In the obese group, Co levels were negatively correlated with FINS levels (r = –0.383; *p* = 0.003) and HOMA-IR (r = –0.332; *p* = 0.011), and positively correlated with QUICKI levels (r = 0.332; *p* = 0.011). Regarding serum Mn levels, a significant negative correlation was found with FG levels (r = –0.313; *p* = 0.021), while the TyG index showed a significant negative correlation with Mn levels (r = –0.271; *p* = 0.048) in the group of overweight women. Likewise, in the group of obese women, a significant positive correlation was identified between Mn levels and age (r = 0.296; *p* = 0.024). However, these Mn-related associations din not remain significant after FDR correction. No statistically significant associations were found between serum Co or Mn levels and the other anthropometric or biochemical variables evaluated (Table 3).

### 3.6. Multiple Linear Regression Analysis

Table 4 presents the results of the multiple linear regression analysis examining the relationship between serum Co levels and IR-related markers in obese women. In both adjusted models, Co was negatively associated with FINS (Model 1: β = −0.373, *p* = 0.003; Model 2: β = −0.363, *p* = 0.006) and HOMA-IR (Model 1: β = −0.361, *p* = 0.003; Model 2: β = −0.339, *p* = 0.010). In turn, a positive association was observed between Co and QUICKI (Model 1: β = 0.351, *p* = 0.004; Model 2: β = 0.320, *p* = 0.015). On the other hand, we also evaluated the association between Mn levels and the TyG index and FG using both models, but these associations did not remain significant.

## 4. Discussion

The interaction between trace elements and insulin metabolism is an emerging area of research in the pathophysiology of obesity and T2DM. Despite its clinical relevance, the role of metals such as Co and Mn has been little explored in human populations. Our study contributes to the understanding of this link by analyzing these associations in overweight and obese Mexican women, groups in which a high prevalence of IR has been documented. Obesity is characterized by alterations in lipid and glucose metabolism, accompanied by increased release of hormones, proinflammatory cytokines, and other mediators involved in the development of IR and progression to T2DM [31].

Our results demonstrate significant anthropometric and metabolic differences between overweight and obese women. These findings confirm that obesity is associated with an altered metabolic profile, characterized by elevated levels of various clinical markers. Specifically, obese participants exhibited increased abdominal adiposity, as reflected by WC, WHR, WHtR, as well as an elevated FINS, a higher HOMA-IR index, and reduced insulin sensitivity indicators, such as QUICKI. Similar alterations in these metabolic parameters have been reported in other studies involving individuals with a BMI greater than 30 kg/m^2^ [32,33]. Additionally, they have reported that, as BMI levels increase, HOMA-IR increases significantly [34]. Interestingly, the study population generally did not exhibit dysglycemia. However, significant changes were observed in FINS levels and specific markers of IR, including HOMA-IR and TyG-BMI. These findings are consistent with those reported in a cohort of overweight and obese adolescents (5–18 years old) [35]. Additionally, Adpn has shown anti-inflammatory properties and the ability to enhance insulin sensitivity. In this context, reduced levels of Adpn are associated with visceral obesity and metabolic disturbances [36]. Consistent with these findings, the present study also identified a significant decrease in Adpn in the obese group compared to the overweight group. On the other hand, it should be noted that the IR indices used were based on cutoffs from different populations, as no thresholds exist for young Mexican women. Their interpretation should be cautious, especially since TyG and TyG-BMI may overlap with anthropometric measures, limiting their independent value.

One of the most relevant findings of our study was the significant inverse association between serum Co levels and IR markers in obese women. Multiple linear regression analysis found that higher serum Co levels were associated with lower FINS and HOMA-IR levels, as well as greater insulin sensitivity (QUICKI). These results align with those of Chen et al., who identified significant negative correlations between blood Co levels and HOMA-IR in adult women [23], suggesting a potential sex-specific effect. Notably, Chen et al. also identified a dose–response relationship and a possible nonlinear association between Co and IR, which implies that the influence of Co on insulin sensitivity may depend on achieving an optimal exposure range [23]. In this context, the median serum Co concentrations in our cohort of overweight and obese women (0.35–0.40 μg/dL, equivalent to 3.5–4.0 μg/L) were higher than the mean blood Co levels reported by Chen et al. (0.22 μg/L in women), which may reflect differences in population characteristics, environmental exposure, or measurement techniques. Despite these differences, the concordance in the direction and significance of the association supports the biological relevance of cobalt in modulating insulin resistance, particularly among women. Similarly, another study has reported a negative association between Co and HOMA-IR in obese children [37]. Likewise, a metallomic study of children and adolescents with obesity and IR reported lower plasma levels of Co-containing proteins in late responders with worse IR [3].

Interestingly, an association between Co exposure and vitamin A metabolism has been described [38]. The physiological functions of vitamin A are primarily mediated by retinoic acid, and its signaling system regulates gene expression involved in hepatic glucose and lipid metabolism, much like insulin [39]. Furthermore, in agreement with our findings, treating dysfunctional preadipocytes with CoPP restored Adpn levels [40]. Similarly, in obese mouse models, CoPP reversed the reduction in Adpn and improved insulin sensitivity [41,42]. In contrast, a study in adults ≥50 years of age (63% women) reported a positive association between Co and insulin levels, regardless of diabetic status [43]. On the other hand, Yang et al. found no association between urinary Co and FG, FINS, or HOMA-IR in a predominantly overweight population [BMI > 25 kg/m^2^] with 43.5% women [44]. Menke et al. also reported no association between urinary Co and HOMA-IR in the general population [45]. Unlike these studies, which were predominantly conducted in mixed populations, our homogeneous cohort of obese women suggests a possible modulating or protective effect of Co on glucose metabolism.

Regarding serum Mn, we observed higher Mn concentrations in obese women compared to overweight women, although the differences were not statistically significant. Nevertheless, these findings align with previous studies in mixed adult populations, where slightly higher Mn levels were also reported in obese individuals [46]. Similarly, elevated Mn concentrations have been observed in obese children, adolescents, and men [37,47,48]. Although we found negative associations between Mn and FG (r = –0.313; *p* = 0.021), as well as the TyG index (r = –0.271; *p* = 0.048), in overweight women, these did not reach statistical significance after adjusting the linear regression model for anthropometric variables. These inverse associations for Mn were limited to the overweight subgroup and should be interpreted cautiously, as they did not persist after adjustment. In line with mechanistic and epidemiological evidence, our interpretation refers to adequate (but not excessive) Mn status supporting glycemic homeostasis within physiological ranges, rather than implying a causal effect or endorsing supplementation. Given the cross-sectional design, these findings do not establish causation and require confirmation in longitudinal or interventional studies. Consistent with a cautious interpretation, a longitudinal study reported lower FG across higher urinary Mn quartiles in men, with weaker associations in women [49]. Also, in the general population (28% with diabetes), an increase in serum Mn levels was associated with a downward trend in FG values or a lower prevalence of prediabetes in older adults [18,43]. On the other hand, experimental evidence in animal models has shown that, in mice fed a high-fat diet, Mn treatment improved glucose tolerance and stimulated insulin secretion by pancreatic islets [50].

Although our findings showed subgroup-specific associations of Co and Mn with insulin resistance markers. In the case of Co, the observed patterns may reflect differences in vitamin B_12_ nutritional status rather than a direct metabolic effect of inorganic Co, since Co is the central atom of cobalamin. Likewise, while adequate Mn is essential for enzymatic antioxidant defense and insulin secretion, excessive exposure is known to be neurotoxic and metabolically harmful. Therefore, our data indicate associations within physiological ranges, and future studies considering vitamin B_12_ intake, diet, and environmental exposures are needed to clarify these mechanisms.

This study has significant limitations. Its cross-sectional design prevents inferring causality between serum levels of Co and Mn and IR; the observed associations should be interpreted as preliminary. The sample size may have been insufficient to detect minor associations, especially in subgroups. Moreover, key factors such as dietary intake, environmental exposure, physical activity, socioeconomic status, or inflammation, which may affect metal concentrations, were not evaluated. Furthermore, our study focused exclusively on young women to minimize variability related to sex and age; however, this approach restricts the extrapolation of our findings to men, older women, or other ethnic groups. Future longitudinal studies should address these limitations to strengthen the evidence on the role of these trace elements in IR.

## 5. Conclusions

To conclude this research, we observed subgroup-specific associations: in women with obesity, serum Co was inversely associated with FINS and HOMA-IR and positively with QUICKI after adjustment; in women with overweight, serum manganese showed negative correlations with FG and TyG that did not persist in adjusted models. These preliminary findings suggest that increased availability of these trace elements could be related to improved IR. However, longitudinal and experimental studies including additional variables and more diverse populations are needed to understand the underlying physiological mechanisms and evaluate the clinical applicability of these trace elements as potential biomarkers or modulators of glucose metabolism.

## Figures and Tables

**Table 1:** Anthropometric and metabolic characteristics of overweight and obese women.

Parameters	Overweight	Obesity	*p* Value
	n = 54	n = 58	
Age (years)	22 (20.75–30.25)	23 (21–32.25)	0.305
Weight (kg)	69.45 (65.38–75.45)	86.80 (80.10–96.60)	**<0.001**
Height (cm)	157.50 (154–161.25)	160 (156–163)	0.056
WC (cm)	91 (86.88–96.25)	106 (99.13–114.25)	**<0.001**
HC (cm)	104 (100.75–107.63)	116.50 (112–123)	**<0.001**
WHR	0.87 (0.85–0.92)	0.91 (0.87–0.95)	**0.004**
WHtR	0.58 (0.55–0.60)	0.66 (0.62–0.71)	**<0.001**
BMI (kg/m^2^)	28.20 (26.61–29.03)	34.14 (32.44–37.16)	**<0.001**
FG (mg/dL)	83.96 (78.10–91.36)	87.56 (79.05–95.77)	0.212
TG (mg/dL)	107.46 (83.10–156.49)	128.10 (87.43–189.53)	0.085
TC (mg/dL)	152.26 (125.26–167.43)	159.86 (142.95–190.43)	0.057
FINS (µU/mL)	15.70 (11.25–21.14)	21.95 (17.50–30.15)	**<0.001**
Adpn (ng/mL)	6.17 (5.07–7.69)	5.15 (3.62–6.81)	**0.011**
HOMA-IR	3.50 (2.19–4.29)	4.71 (3.71–6.94)	**<0.001**
QUICKI	0.32 (0.31–0.34)	0.30 (0.29–0.31)	**<0.001**
TyG index	4.58 (4.45–4.73)	4.63 (4.47–4.83)	0.055
TyG-BMI	127.61 (121.02–135.14)	162.03 (149.84–172.09)	**<0.001**
Co (μg/dL)	0.35 (0.23–0.58)	0.40 (0.32–0.59)	0.237
Mn (μg/dL)	0.20 (0.14–0.35)	0.30 (0.16–0.46)	0.136

Data expressed as median (25th–75th percentile). Differences between the overweight and obese groups were analyzed using the Mann–Whitney U test. Abbreviations: WC, waist circumference; HC, hip circumference; WHR, waist-to-hip ratio; WHtR, waist-to-height ratio; BMI, body mass index; FG, fasting glucose; TG, triglycerides; TC, total cholesterol; FINS, fasting insulin; Adpn, adiponectin; HOMA-IR, homeostasis model assessment of insulin resistance; QUICKI, quantitative insulin sensitivity check index; TyG, triglyceride–glucose index; TyG-BMI = BMI × TyG index. Significant *p*-values (*p* < 0.05) are shown in bold.

**Table 2:** Characteristics of the study sample in relation to markers of insulin resistance.

Parameters	Reference Value	Overweight		Obesity	
		n	%	n	%
WC (cm)	≥88	36	66.67	58	100
FG (mg/dL)	≥100	4	7.41	9	15.52
FINS (µU/mL)	≥25	8	14.81	24	41.38
HOMA-IR	≥2.5	38	70.37	57	98.28
QUICKI	≤0.33	37	68.52	56	96.55
TyG index	≥4.68	17	31.48	25	43.1
TYG-BMI	≥227	0	0	2	3.45

Abbreviations: WC, waist circumference; FG, fasting glucose; FINS, fasting insulin; HOMA-IR, homeostasis model assessment of insulin resistance; QUICKI, quantitative insulin sensitivity check index; TyG, triglyceride–glucose index; TyG-BMI = BMI × TyG index.

**Table 3:** Correlation between anthropometric variables and biochemical markers associated with insulin risk and serum levels of Co and Mn.

Parameters	Co (μg/dL)		Mn (μg/dL)	
	Overweight	Obesity	Overweight	Obesity
Age (years)	−0.052 (0.708)	−0.169 (0.206)	−0.019 (0.894)	0.296 (*0.024)
Weight (kg)	−0.154 (0.265)	−0.171 (0.200)	−0.023 (0.866)	−0.074 (0.579)
Height (cm)	−0.119 (0.391)	0.000 (0.999)	−0.097 (0.485)	−0.058 (0.666)
WC (cm)	−0.270 (*0.048)	0.054 (0.689)	−0.089 (0.523)	−0.011 (0.935)
HC (cm)	−0.181 (0.190)	−0.164 (0.217)	0.034 (0.804)	0.076 (0.571)
WHR	−0.188 (0.174)	0.163 (0.220)	−0.154 (0.268)	−0.070 (0.602)
WHtR	−0.257 (0.060)	0.062 (0.642)	−0.058 (0.677)	−0.016 (0.905)
BMI (kg/m^2^)	−0.120 (0.389)	−0.202 (0.133)	0.071(0.610)	−0.048 (0.726)
FG (mg/dL)	0.259 (0.059)	**0.076 (0.570)**	−0.313 (*0.021)	0.101 (0.450)
TG (mg/dL)	0.048 (0.730)	0.135 (0.311)	−0.203 (0.142)	0.036 (0.786)
TC (mg/dL)	−0.019 (0.891)	−0.025 (0.854)	0.024 (0.864)	0.059 (0.660)
FINS (µU/mL)	−0.047 (0.734)	**−0.383 (0.003)**	−0.152 (0.272)	−0.229 (0.084)
Adpn (ng/mL)	0.119 (0.390)	−0.027 (0.838)	0.107 (0.441)	0.117 (0.383)
HOMA-IR	−0.082 (0.557)	**−0.332 (0.011)**	−0.202 (0.143)	−0.137 (0.306)
QUICKI	0.082 (0.557)	**0.332 (0.011)**	0.202 (0.143)	0.137 (0.306)
TyG index	−0.014 (0.918)	0.131 (0.327)	−0.271 (*0.048)	0.071 (0.594)
TyG-BMI	−0.085 (0.540)	−0.133 (0.323)	−0.077(0.580)	−0.043 (0.751)

Data are expressed as Spearman’s Rho (*p*-value). Abbreviations: WC, waist circumference; HC, hip circumference; WHR, waist-to-hip ratio; WHtR, waist-to-height ratio; BMI, body mass index; FG, fasting glucose; TG, triglycerides; TC, total cholesterol; FINS, fasting insulin; Adpn, adiponectin; HOMA-IR, homeostasis model assessment of insulin resistance; QUICKI, quantitative insulin sensitivity check index; TyG, triglyceride–glucose index; TyG-BMI = BMI × TyG index. Significant *p*-values (*p* < 0.05) are shown in bold. * *p*-values no longer remained significant after Benjamini–Hochberg correction (FDR = 0.01).

**Table 4:** Association between serum Co and markers of insulin resistance in obese women.

Parameters	Model 1			Model 2		
	B (95% CI)	β	*p* Value	B (95% CI)	β	*p* Value
FINS	−0.299 (−0.475, −0.106)	−0.373	**0.003**	−0.284 (−0.484, −0.085)	−0.363	**0.006**
HOMA-IR	−0.287 (−0.474, −0.099)	−0.361	**0.003**	−0.271 (−0.475, −0.067)	−0.339	**0.010**
QUICKI	0.011 (0.004, 0.018)	0.351	**0.004**	0.010 (0.002, 0.018)	0.320	**0.015**

Data are expressed as unstandardized B and as β value (95% CI). The following variables were natural log-transformed before analysis: FINS, HOMA-IR, QUICKI, WC, HP, BMI, and serum Co. Abbreviations: FINS (µU/mL), fasting insulin; HOMA-IR, homeostasis model assessment of insulin resistance; QUICKI, quantitative insulin sensitivity check index. Multiple linear regression analysis. Model 1: adjusted for height, WC, and HP; Model 2: adjusted for WHR and BMI. Significant *p* values (<0.05) are represented in bold. All *p*-values remained significant after Benjamini–Hochberg correction (FDR = 0.05).

## Data Availability

The data presented in this study are available on request from the corresponding author.
